# Liver organoid culture methods

**DOI:** 10.1186/s13578-023-01136-x

**Published:** 2023-11-01

**Authors:** Yiqing Hu, Xiaoyi Hu, Jia Luo, Jiacheng Huang, Yaohan Sun, Haoyu Li, Yinbiao Qiao, Hao Wu, Jianhui Li, Lin Zhou, Shusen Zheng

**Affiliations:** 1 Division of Hepatobiliary and Pancreatic Surgery, Department of Surgery, The First Affiliated Hospital, Zhejiang University School of Medicine, Hangzhou, 310003 China; 2 NHC Key Laboratory of Combined Multi-Organ Transplantation, Hangzhou, 310003 China; 3 Department of Hepatobiliary and Pancreatic Surgery, Shulan (Hangzhou) Hospital, Zhejiang Shuren University School of Medicine, Hangzhou, 310015 China; 4 The Organ Repair and Regeneration Medicine Institute of Hangzhou, Hangzhou, 310003 China; 5 Jinan Microecological Biomedicine Shandong Laboratory, Jinan, 250117 China

**Keywords:** Liver organoid, Hepatic organoid, Culture method, Medium components

## Abstract

Organoids, three-dimensional structures cultured in vitro, can recapitulate the microenvironment, complex architecture, and cellular functions of in vivo organs or tissues. In recent decades, liver organoids have been developed rapidly, and their applications in biomedicine, such as drug screening, disease modeling, and regenerative medicine, have been widely recognized. However, the lack of repeatability and consistency, including the lack of standardized culture conditions, has been a major obstacle to the development and clinical application of liver organoids. It is time-consuming for researchers to identify an appropriate medium component scheme, and the usage of some ingredients remains controversial. In this review, we summarized and compared different methods for liver organoid cultivation that have been published in recent years, focusing on controversial medium components and discussing their advantages and drawbacks. We aimed to provide an effective reference for the development and standardization of liver organoid cultivation.

## Introduction

Organoids are multicellular three-dimensional (3D) structures that mimic the tissue architecture and function to some extent [1] and effectively recapitulate the micro-environment and cell–cell interactions observed in vivo [2]. In recent years, experts have gradually reached a consensus on the definition of an organoid: a 3D structure that is derived from stem cells, progenitor cells, or differentiated cells and is capable of recapitulating certain functions and the architecture of the native tissue in vitro [3, 4]. Epithelial organoids originating from pluripotent stem cells (PSCs) or primary liver tissue (intrahepatic cholangiocytes, extrahepatic cholangiocytes, and hepatocytes) can be considered liver organoids [4].

So far, the limited availability of human samples as well as the lack of suitable in vitro models that accurately simulate the physiological situation have become the major obstacles to advances in research on chronic liver disease [5]. Although numerous cell lines and animal models have been applied in researches, there is still a need to pursue research models that mimic the in vivo niche. Currently, the limitations of the widely applied research models remain concerning. Animal models are costly and constrained by interspecific differences and ethical issues [6], while two-dimensional (2D) cell cultures are unable to replicate cellular heterogeneity or the complex architecture of organs [7]. In late December 2022, the Food and Drug Administration (FDA) issued “*FDA Modernization Act 2.0”*, declaring that new medicines no longer need to be tested in animals before human drug trials and encouraging the exploration of alternative models [8]. Researchers and pharmaceutical companies are urgently looking for nonanimal methods to study pharmacokinetics and drug toxicity [9]. Since the liver is the main detoxification organ and metabolic organ in the human body, liver organoids may become an ideal choice. Furthermore, because of their ability to structurally and functionally imitate their tissues of origin, organoids have been established as state-of-the-art instruments for human liver biology research in the context of both health and disease [10]. In addition, liver organoid technology provides a suitable platform for research on liver development, complex diseases, therapeutic transplantation, etc. [1, 11].

Notably, there are variations in the current culture procedures used for liver organoids in published studies, with differences in culture methods, cell sources, and most significantly, culture medium components. Although Hunch previously published a protocol to cultivate human and mouse adult liver 3D organoids [12], we found it confusing that numerous scholars used contrasting medium schemes to generate organoids from the same cell source [13, 14]. In addition, liver organoids derived from human PSCs, which have an extensive research history, have also been cultured in a variety of different media [15–17]. With the quick development of organoid systems and the rapid increase in the complexity of tissue components, the standardization and validation of organoid systems are urgently needed. The unification of a standard culture condition would be beneficial for clinical application, mass production, and the establishment of a quality inspection system for organoids [4]. To date, a series of industry standards have been introduced in the field of gastrointestinal organoids and tumor organoids in China [18, 19], but there is still a lack of regulation of liver organoids.

To address this issue, we summarized the current culture methods for liver organoids and focused on the effects of different medium components on the growth, proliferation, and differentiation of tissue-derived liver organoids. We hoped to determine the effects of different ingredients on organoids, which would help define reproducible culture conditions and facilitate the establishment of standard culture criteria for liver organoids.

## Cells sources for organoid generation

### Liver organoids derived from PSCs

PSCs have infinite proliferation potential and can differentiate into all three embryonic germ layers (endoderm, mesoderm, and ectoderm) [20], which allows them to form well-functioning liver organoids. Embryonic stem cells (ESCs) and induced pluripotent stem cells (iPSCs) are currently the most common cell sources for PSC-derived organoids [21]. ESCs are isolated from the inner cell mass (ICM) of the blastocyst [22] whereas iPSCs are artificial [23].

PSCs have significant advantages in organoid formation. Human ESCs/iPSCs have become an attractive cell source for organoids because of their high pluripotency, plasticity, and infinite proliferation capacity. These features enable ESCs/iPSCs to differentiate into viable and functional hepatocyte-like cells in the presence of specific signaling factors [24, 25]. iPSCs can be generated from unlimited sources with different genetic backgrounds [26] and are not associated with ethical concerns (iPSCs are not derived from human embryos [27]), in contrast to ESCs.

**Fig. 1:**
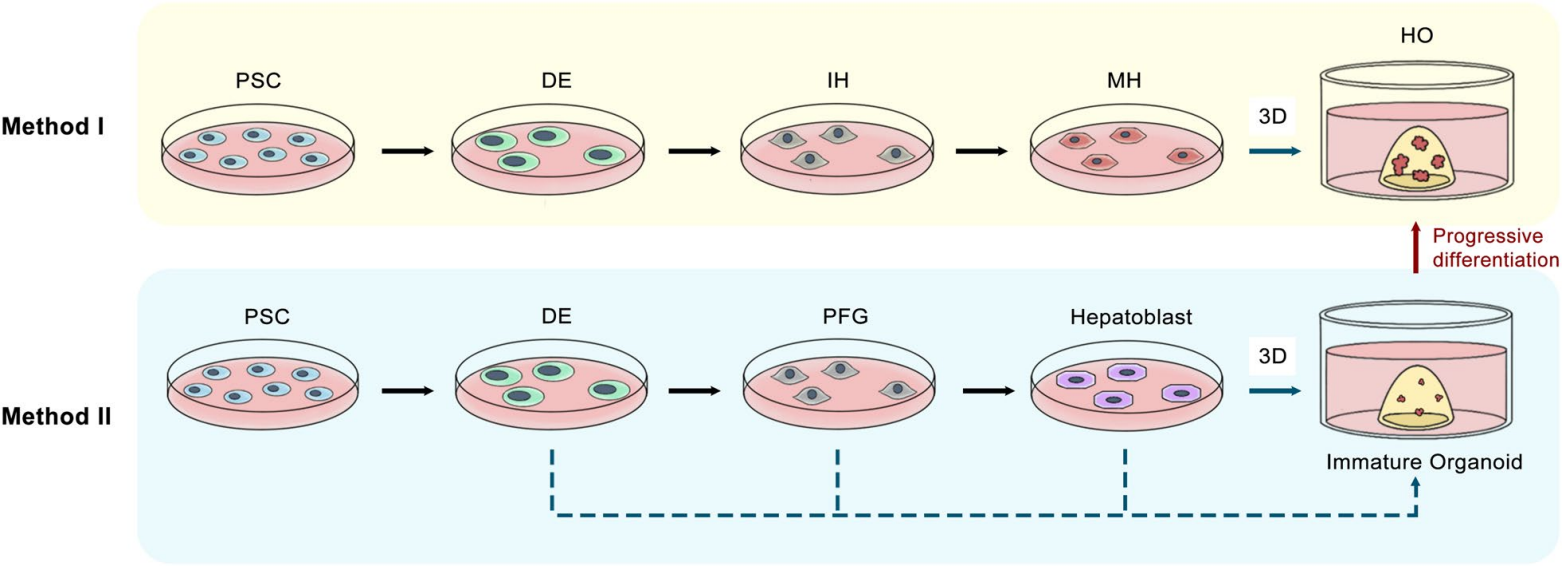
Mainstream methods for generating PSC-derived liver organoids. Method I: Organoids are formed on the basis of PSC-induced mature hepatocytes; Method II: Spheroids are formed during the process of PSC differentiation, and immature spheroids are then further differentiated into mature hepatic organoids. *PSCs* pluripotent stem cells, *DE* definitive endoderm, *IHs* immature hepatocytes, *MHs* mature hepatocytes, *Hos* hepatic organoids, *PDF* posterior foregut

It has been verified that iPSCs can be induced to differentiate into liver organoids in a stepwise manner via manipulation of the anterior–posterior gradients of specific factors [15]. Here we summarized the commonly used cultivation approaches for PSC-derived organoids and classified them into two general types. *I.* The initial generation of mature hepatocytes from iPSCs and the subsequent generation of organoids via 3D culture [15, 28, 29]. After achieving definitive endoderm (DE) differentiation in Activin A rich medium, factors including bone morphogenetic protein (BMP), fibroblast growth factors (FGFs), and dexamethasone are used to achieve the differentiation of hepatic endoderm (HE), immature hepatocytes (IHs, also known as the hepatoblast), and then mature hepatocytes (MHs). After MHs are obtained, 3D culture and further differentiation are performed, and liver organoids are eventually obtained [15]. *II.* Spheroids were produced at an earlier stage of the differentiation process and then further differentiated immature spheroids into mature liver organoids [16, 30–33]. For example, some studies reported the formation of spheroids after the induction of the posterior foregut (PFG) [16, 30, 33]. Others also reported the formation of spheroids at the DE stage [31] or the hepatoblasts stage [32]. Both methods can successfully produce liver organoids that can self-renew and maintain hepatic characteristics during long-term culture (Fig. 1).

Human PSC-derived liver organoids are now recognized as one of the leading in vitro model systems for disease treatment and drug cytotoxicity testing on a large scale. Organoids cultured from PSCs have the advantages of unlimited proliferation, the ability to generate different cell types and suitability for genome editing [34]. PSC-derived liver organoids were first generated by Takebe et al., and the engraftment of these transplanted iPSC organoids onto the mesentery was shown to successfully rescue ganciclovir-induced liver failure [35]. Since then, numerous studies have used iPSC organoids as a tool for transplantation and studying hepatic diseases [28, 33, 36]. Moreover, Shinozawa and colleagues developed a toxicity screening system based on iPSC-derived liver organoids, and this system exhibited high predictive value for testing 238 marketed drugs [33].

However, epigenetic and genetic aberrations can occur during the derivation and reprogramming of PSC-induced liver organoids [37]. In contrast to the high genome stability of hepatic progenitor cells (HPCs) isolated from liver tissue during the amplification process, PSC-induced organoids exhibit both chromosome and aneuploidy alterations [38]. In addition, hepatocyte-like cells induced from iPSCs still have deficits in functional maturity and genome stability, and the artificial culture conditions do not fully resemble the natural environment, which impacts the function of iPSC-derived hepatocytes [25]. Furthermore, the high economic cost of iPSC derivation and expansion is another important obstacle [39]. All of these factors will unfortunately limit the application of liver organoids derived from PSCs in regenerative medicine.

### Liver organoids derived from liver tissue

Quiescent liver stem cells are thought to reside in the bile ducts (the Canal of Hering) [40]. Therefore, the first tissue-derived liver organoid was established successfully by the Clevers team using biliary duct fragments and sorted Lgr5^+^ cells from mice [41]. Since then, scholars have made efforts to culture liver organoids from the biliary tree [13], sorted bile duct cells [42] and bile [43], etc. In recent years, much progress has been made in establishing liver organoids directly from mature hepatocytes as well [44–47].

Primary hepatocytes have now become one of the main sources of liver organoids. Increasing numbers of studies have revealed that mature hepatocytes still have stemness potential and proliferation abilities in specific environments. Former research proved that chronically injured mature hepatocytes can be reprogrammed into HPCs and subsequently facilitate hepatocyte mass reconstruction [48]. Lineage tracing also confirmed the presence of hepatocyte-derived progenitor cells [49, 50]. In addition, a recent study by Wang et al. defined “proliferating human hepatocytes (ProliHHs)” as dedifferentiated primary human hepatocytes exhibiting both hepatocyte and progenitor characteristics [51].

Primary tissue-derived organoids are more mature and have higher genome stability than those induced from PSCs, making the direct cultivation of primary liver cells into organoids an attractive approach [42]. Hunch and coworkers verified that organoids expanded from primary ductal cells retained their phenotypic and genetic stability during the long-term in vitro culture process [42]. In addition, when culture was initiated from hepatocytes, it was noted that these organoids could better imitate the regenerative response after human partial hepatectomy, such as strong upregulation of albumin and cytochrome expression [45]. However, it is also worth pointing out that the long-term proliferative capacity of mature human hepatocyte organoids appears to be limited compared to that of fetal human hepatocytes or adult mouse primary hepatocytes [45]. Until now, the culture of adult human hepatocyte organoids has remained challenging.

**Fig. 2:**
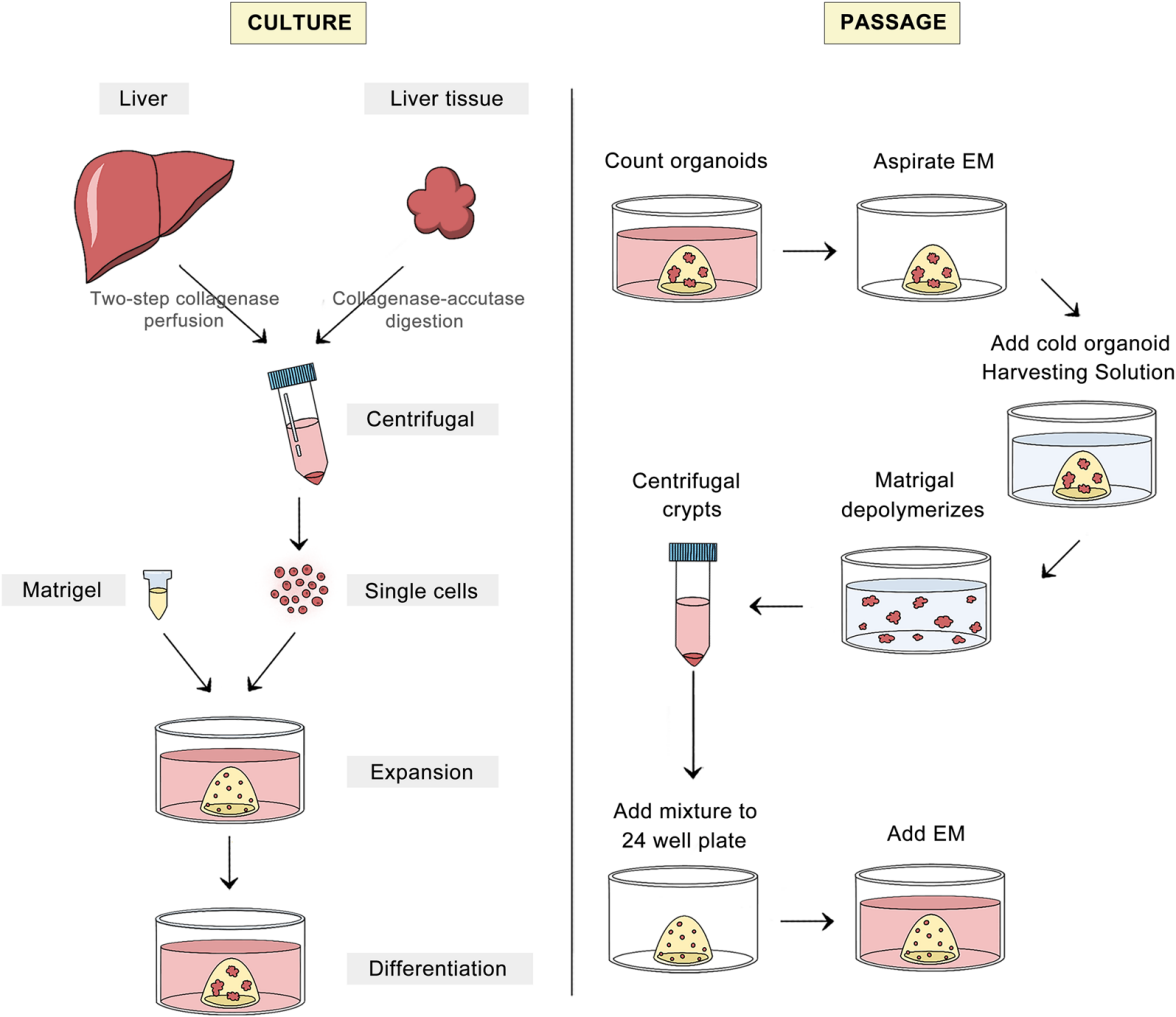
The culture and passage procedure for tissue-derived liver organoids. Liver or liver tissues are digested into single cells and then mixed with Matrigel for seeding in plates, and medium is added for culture. For passage, after removing the medium, harvesting solution is added to decompose the Matrigel, and organoid fragments are mixed with fresh Matrigel and seeded. *EM* expansion medium

The high genetic stability of tissue-derived liver organoids as well as their high level of similarity to their organs of origin make them attractive choices for ex vivo testing and new therapeutic applications. Therefore, despite the difficulties of maintaining proliferative capacity and metabolic function during long-term expansion ex vivo [52], tissue-derived organoids possess high research value and broad prospects in clinical applications. Here, we summarized the specific processes used for the culture of liver organoids from primary liver tissue (Fig. 2):

I. Hepatocyte organoids: The liver tissue is dissociated into single cells (to obtain primary hepatocytes from mice by two-step collagenase perfusion [53] or from human liver biopsies by collagenase-acutase digestion [54]) and then resuspended in Matrigel after centrifugation [45].
II. Cholangiocyte organoids: (i) Cystic organoids are directly induced from isolated biliary duct fragments [13]. (ii) Lgr5^+^ or EpCAM^+^ ductal cells are sorted from primary liver cells obtained by collagenase perfusion to form organoids [42]. (iii) All primary liver cells are embedded in Matrigel, and cells are then cultured in under specific media to direct them toward a duct fate [14].

In the current study, most investigators used the following embedding method to seed these tissue and cell sources to form liver organoids: The Matrigel/cell mixture is seeded in 24-well plates at 50 μl per well to enable the formation of dome-shaped structures. Incubation in the cell incubator (37 ℃) for 15 min is required for Matrigel polymerization. After solidification, 500 μl of specific medium is added and later renewed at specific intervals. Passage can be performed after approximately 14 days, with the organoids enzymatically or mechanically fragmented and reseeded in new Matrigel (Fig. 2). For differentiation, after 7–10 days of amplification, the original medium is replaced with the same volume of differentiation medium, followed by incubation for 11–13 days [12]. The detailed ingredients of those media are elaborated in detail below.

## The application of Matrigel

During 3D culture, a suitable extracellular matrix (ECM) plays an indispensable role in providing structural support for cells [55]. As early as 1977, a murine basement-membrane-producing tumor that produced ECM in large quantities was identified [56]. These tumors were then designated Engelbreth-Holm-Swarm (EHS) tumors, and their extracts can be processed (by adding heparan sulfate proteoglycan and type IV collagen under physiological conditions) into gel-like structures that were analogous to the basement membrane [57]. Since then, the composition of the generated substance has been further developed, and it was finally commercialized as ‘Matrigel’. The main components of Matrigel now include laminin (a major component), collagen IV, heparan sulfate proteoglycans, entactin/nidogen, and numerous growth factors [58].

Matrigel has long been applied for the culture of various cell types and is used to form different kinds of 3D organoids, such as gut organoids, liver organoids, brain organoids, retinal organoids, and kidney organoids [59]. However, despite its apparent advantages as a useful tool, the limitations of Matrigel cannot be ignored: (i) complex and changeable components [60]; (ii) biochemical and mechanical variations within or across batches [61, 62]; and (iii) the potential for antigenicity (xenogeneic contaminants or viral contaminants) [63]. These factors have led to a lack of reproducibility and stability in organoid culture experiments using Matrigel.

Recently, with the development of material science, numerous natural and synthetic alternatives to Matrigel have emerged. The natural alternatives consist of natural hydrogels and decellularized ECM [64]. Natural hydrogels are based on natural polysaccharides (e.g., alginate, hyaluronic acid and chitosan) and proteins (e.g., collagen, gelatin and fibrin), while decellularized ECM can be obtained from organs or tissues [64] (e.g., ECM obtained from decellularized liver tissue supports the growth of cholangiocyte organoids [65]). The similarity of these alternatives to native ECM makes them suitable for cell growth and differentiation and researchers have widely applied them in the culture of organoids [65–67], but the disadvantages include low stability and rapid degradation [64]. Regarding synthetic alternatives, synthetic hydrogels derived from polyethylene glycol (PEG) and its derivatives emerged to support the culture of different organoids [68–70]. The highly tunable physicochemical, mechanical and biological parameters of synthetic hydrogels are crucial for controlled and reproducible organoid formation [71]. However, screening scaffolds with suitable parameters to direct cellular behaviour is highly time- and cost-consuming, and synthetic scaffolds face challenges in recapitulating the intricate fibre-like architecture of native tissues [63]. Currently, researchers continue to use Matrigel for liver organoid culture due to its accessibility, convenience, and versatility.

## Culture media for tissue-derived liver organoids

Liver organoid systems require a carefully composed culture environment. In this review, we focused on the culture media for liver organoids derived from liver tissue. The Clevers group first identified expansion medium (EM) and differentiation medium (DM) as the fundamental media for liver organoids [41]. EM allowed tissue fragments/sorted cells to rapidly divide and grow into 3D structures; however, these 3D structures had bipotential and expressed both bile duct and hepatocyte-lineage markers, accompanied by a loss of mature hepatocyte markers. Therefore, DM was added to ultimately induce a hepatocyte fate [41]. Although attempts have been made in subsequent studies to add or remove some factors on the basis of “EM” and “DM” to generate different types of liver organoids, these two media have continued to be the mainstream. The functions of the main ingredients in these culture media are summarized in Table 1 and will be further discussed below.

**Table 1:** Functions of organoid culture medium components

Ingredient	Belong	Property	Pathway	Functions in liver organoid culture
AdDMEM/F12	EM, DM	Culture medium	N/A	Basal medium
William’s E medium	EM, DM	Culture medium	N/A	Basal medium
HEPES [72]	EM, DM	Additive	N/A	PH stabilizer
GlutaMax [73]	EM, DM	Additive	N/A	Mammalian cell culture
Antibiotics	EM, DM	Additive	N/A	Antibacterial
Non-Essential Amino Acids [74]	EM, DM	Additive	N/A	Cell growth and viability
B-27 [75]	EM, DM	Additive	N/A	Proliferation and differentiation of stem cells
N_2_ [75]	EM, DM	Additive	N/A	Proliferation and differentiation of stem cells
Wnt 3a [76]	EM	Cytokine	Wnt signaling	Hepatocyte proliferation
R-spondin 1 [77, 78]	EM, DM	Cytokine	Wnt signaling	Stem cell maintenance and expansion
Noggin [78]	EM	Cytokine	BMP signaling	Stem cell expansion
EGF [78]	EM, DM	Cytokine	EGFR signaling	Cell proliferation
TGFα [79]	EM	Cytokine	EGFR signaling	Cell proliferation
HGF [80]	EM, DM	Cytokine	HGF signaling	Hepatocyte proliferation
FGFs [42, 45]	EM, DM	Cytokine	FGF signaling	FGF7, FGF10: hepatocyte proliferation FGF19: hepatocyte differentiation
BMP7 [81, 82]	DM	Cytokine	BMP signaling	Hepatocyte proliferation and differentiation
TNFα [83]	EM, DM	Cytokine	TNF signaling	Hepatocyte proliferation
OSM [84]	DM	Cytokine	OSM signaling	Human hepatocyte differentiation
N-acetylcysteine [85, 86]	EM, DM	Small molecule compound	TGF-β signaling	Antioxidant Hepatocyte viability and function
Nicotinamide [41]	EM, DM	Small molecule compound	N/A	Organoid formation and long-term culture
Gastrin I [87, 88]	EM, DM	Small molecule compound	Gastrin signaling	Organoid long-term culture
CHIR99021 [89, 90]	EM, DM	Small molecule compound	Wnt signaling	Stem cell maintenance
A83-01 [42]	EM, DM	Small molecule compound	TGF-β signaling	Organoid formation
Y-27632 [45, 46, 89]	EM	Small molecule compound	ROCK signaling	Stem cell expansion and maintenance Prevent anoikis
FSK [42]	EM	Small molecule compound	cAMP signaling	Stem cell maintenance Human organoid long-term culture
Dexamethasone [45, 91]	DM	Small molecule compound	Notch signaling	Hepatocyte differentiation
DAPT [92]	DM	Small molecule compound	Notch signaling	Hepatocyte differentiation

### Expansion medium

Here, we summarize the ingredients of different expansion media from published articles that reported the successful cultivation of liver organoids in the past 10 years. It was observed that the vast majority of these constituents were found to be used differently (Table 2), and the function of each ingredient is provided in Table 1 for reference.

**Table 2:** Detailed components of different expansion media

Origin	2013 Huch et al. [41]	2015 Huch et al. [42]	2016 Broutier et al. [12]		2018 Peng et al. [44]	2018 Hu et al. [45]		2020 Sorrentino et al. [13]		2020 Gómez-Mariano et al. [14]	2021 Hendriks et al. [46]	2023 Hendriks et al. [93]
	Mouse	Human	Mouse	Human	Mouse	Mouse	Human	Mouse	Human	Human	Human	Human
Type	Cholangiocyte organoid	Cholangiocyte organoid	Cholangiocyte organoid	Cholangiocyte organoid	Primary hepatocyte organoids	Hepatocyte organoids	Fetal hepatocyte organoids	Cholangiocyte organoid	Cholangiocyte organoid	Cholangiocyte organoid	Fetal hepatocyte organoids	Fetal hepatocyte organoids
Basal medium	AdDMEM/F12	AdDMEM/F12	AdDMEM/F12	AdDMEM/F12	William’s E medium	AdDMEM/F12	AdDMEM/F12	AdDMEM/F12	AdDMEM/F12	AdDMEM/F12	AdDMEM/F12	AdDMEM/F12
HEPES (mM)	/	/	10	10	/	√	√	/	/	10	10	10
GlutaMax	/	/	1%	1%	1%	√	√	/	/	1%	1%	1%
Antibiotics	/	/	Penicillin–Streptomycin: 1%	Penicillin–Streptomycin: 1%	Penicillin–Streptomycin: 1% Normocin: 0.2%	√	√	/	/	Penicillin–Streptomycin: 1%	Penicillin–Streptomycin: 100 U/ml Primocin: 50 ug/ml	Penicillin–Streptomycin: 100 U/ml Primocin: 50 ug/ml
Non-Essential Amino Acids	Contained in AdDMEM/F12	Contained in AdDMEM/F12	Contained in AdDMEM/F12	Contained in AdDMEM/F12	1%	Contained in AdDMEM/F12	Contained in AdDMEM/F12	Contained in AdDMEM/F12	Contained in AdDMEM/F12	Contained in AdDMEM/F12	Contained in AdDMEM/F12	Contained in AdDMEM/F12
B-27	√	1%	2%	2%	2%	√	√	√	√	/	2%	2%
N_2_	√	1%	/	1%	1%	/	/	√	√	/	/	/
N-acetylcysteine (mM)	1.25 μM	1.25	1	1	1.25	1.25	1.25	1.25 μM	1.25 μM	1	1.25	1.25
Rspo1	RSPO1: 1 μg/ml or RSPO1 CM^a^: 10%	RSPO1 CM^a^: 10%	RSPO1 CM^a^: 5%	RSPO1 CM^a^: 10%	/	RSPO1 CM^a^: 15%	RSPO1 CM^a^: 15%	RSPO1: 1 μg/ml	RSPO1: 1 μg/ml	RSPO1 CM^a^: 5%	RSPO1 CM^a^: 15%	RSPO1 CM^a^: 15%
Nicotinamide (mM)	10	10	10	10	10	10	10	10	10	10	2.5	2.5
Gastrin I (nM)	10	10	10	10	/	10	10	10	10	10	10	10
EGF (ng/ml)	50	50	50	50	25	50	50	50	50	50	50	50
HGF (ng/ml)	50	25	50	25	50	25	50	50	25	50	50	50
FGFs (ng/ml)	FGF10: 100	FGF10: 100	FGF10: 100	FGF10: 100	/	FGF10: 50 FGF7: 50	FGF10: 100 FGF7: 100	FGF10: 100	FGF10: 100	FGF10: 100	FGF10: 50 FGF7: 50	FGF10: 50 FGF7: 50
CHIR99021 (μM)	/	/	/	/	3	3	3	/	/	/	3	3
A83-01 (μM)	/	5	/	5	1	1	2	/	5	/	1	1
Noggin (ng/ml)	100^b^	25^b^	25^b^ or noggin CM^a^: 5%	25^b^ or noggin CM^a^: 5%	50^c^	/	/	100^b^	100^b^	25^b^	/	/
Wnt 3a	Wnt 3a CM^ab^	Wnt 3a CM^a^: 30%^b^	Wnt 3a CM^a^: 30%^b^	Wnt 3a CM^a^: 30%^b^	/	/	/	Wnt 3a: 1 μg/ml^b^	Wnt 3a: 1 μg/ml^b^	Wnt 3a CM^a^: 30%^b^	/	/
Y-27632 (μM)	/	10^b^	10^b^	10^b^	10^b^	10^b^	10^b^	10^b^	10^b^	10^b^	50^b^	5-10^bd^
TGFα (ng/ml)	/	/	/	/	/	/	20	/	/	/	20	20
TNFα (ng/ml)	/	/	/	/	100	/	/	/	/	/	/	/
FSK (μM)	/	10	/	10	/	/	/	/	10	/	/	/

^a^Conditioned medium (CM)

^b^Treatment in the first 3–4 days after seeding

^c^Treatment during long-term culture

^d^Extra Y-27632 (10 μM) to minimize anoikis

The effects of the culture additives are reflected on the basal cell culture conditions, and some minor adjustments were reported to be acceptable (Table 2). HEPES, being a zwitterionic organic buffer with low permeability to cell membranes, is routinely included in cell culture medium as a pH stabilizer [72]. Primocin is a primary cellular antibacterial agent, and has been increasingly used in conjunction with Penicillin–Streptomycin to protect primary organoids from microbial contamination [46, 93]. B27 and N2 supplement are both serum-free additives optimized for the culture of neuronal cells and stem cells [75].

The application of cytokines and small molecule compounds is considerably more complex (Table 2). A83-01 is a specific inhibitor of transforming growth factor-β (TGF-β), it was initially added to EM by Huch in an exploration of media for human liver organoid culture based on the mouse liver organoid culture system [42]; moreover, A83-01 was also proven to promote the proliferation of mouse liver organoids in an article by Hu et al. [45].

The Wnt agonist R-spondin 1 is a ligand for Lgr5 [94], and Lgr5 + cells express features of bipotent progenitors in the liver. Initially believed to induce crypt proliferation [95], noggin was then proven to facilitate the expansion of resident stem cells from the bile duct in conjunction with R-spondin 1 [96]. Y-27632 is a Rho-associated kinase (ROCK) inhibitor, and the addition of Y-27632 promotes the proliferation of liver stem/progenitor-like cells [97]. Furthermore, extra Rock inhibitor has been reported to be applied to prevent anoikis [46]. Since the earliest organoid that we now defined was generated, Clevers and colleagues have added epidermal growth factor (EGF), R-spondin 1, noggin, and Y-27632 to the medium [98]. Then, also reported by the Clevers team, gastric organoid was generated soon after [87], and the culture medium was prepared as described with some modifications, including the addition of B27, N2 supplement, N-acetylcysteine, gastrin, FGF10, and Wnt3a. The culture conditions for liver organoids initially resembled these previously defined organoid culture conditions [87, 98], as Clevers believed that the same Lgr5 + stem cell marker would allow those factors to perform similar effects on liver progenitors [41], and additional hepatocyte growth factor (HGF) and nicotinamide were added because of their pro-proliferative effects on hepatocytes [80].

In addition, it was reported that the combination of Y-27632, A-83–01, and CHIR99021 mediates the transformation of mature hepatocytes into liver progenitors in vitro [89], which may account for the usage of CHIR99021 in organoid cultures from primary hepatocytes [44–46, 93]. Notably, since noggin, Wnt3a, and Y-27632 have often been supplied only in the first 3–4 days of culture for the establishment of cholangiocyte organoids, some reports defined the medium containing these three factors as “initial medium” (sometimes referred to as “isolation medium” [12]), which is distinct from the EM that replaces it after the initial stage.

Transforming growth factor alpha (TGFα) belongs to the EGF family of mitogens and shares the same receptor as EGF. Hu et al. demonstrated that the addition of TGFα to mouse hepatocyte organoid cultures can promote the expansion of human fetal hepatocyte organoids [45]. Since then, TGFα has been used frequently in the EM for fetal hepatocyte organoids [46, 93].

Hereafter, the forskolin (FSK) and FGF families, which have controversial application conditions, were chosen for further analysis, and the effects of the recently concerned tumor necrosis factor-α (TNFα) will also be elaborated.

#### FSK

FSK is produced by the roots of *Coleus forskohlii* (an Indian plant) [99] and is known as an activator of cyclic adenosine monophosphate (cAMP) signaling [100]. cAMP was identified by Sutherland and Rall in 1958 and is an essential biological molecule for signaling within and between cells [101]. Since the cAMP pathway participates in numerous metabolic reactions and cell functions [102], its role in cell growth and proliferation has been well recognized [103].

Early studies reported the ability of cAMP to promote hepatocyte proliferation [104, 105]. Rixon and Whitfield explored the interactions among cAMP, DNA replication, and hormones during promitosis of regenerating liver cells and elucidated the possible role of cAMP in the early stages of hepatocyte proliferation [104]. Meanwhile, Hiroyuki et al. proved that prostaglandins (PGs), cAMP agonist, promote the proliferative ability of hepatocytes after partial hepatectomy [106]. Additionally, FSK, which acts as a cAMP agonist, has been reported to independently activate the proliferation of cholangiocytes by increasing cAMP levels through the PKA/Src/MEK/ERK1/2 pathway, and bile secretion and bicarbonate concentrations were also significantly increased [107]. These distinct roles of FSK in cholangiocytes may explain its use in the culture of cholangiocyte organoids [12, 13, 42, 46].

It has been previously reported that FSK induces rapid swelling of intestinal organoids originating from both humans and mice [108]. Concerning liver organoids, FSK is now used in some culture media for liver organoids of human origin. Meritxell Huch initially introduced FSK into the EM for liver organoids culture because the formerly used mouse liver medium failed to support the growth of human liver organoids, and FSK upregulated the gene expression of KRT19 (a ductal marker) and Lgr5 while downregulating that of ALB and CYP3A4 [42]. We accordingly hypothesized that FSK may allow organoids to exhibit more stem cell features. The organoids treated with FSK showed no difference in colony formation but a significant improvement in expansion efficiency during long-term passaging (> 15 months). The authors also found that the removal of FSK resulted in rapid deterioration of organoids in culture, and similar results were observed with other cAMP agonists [42]. Subsequently, more researchers used this valuable factor in the EM for human cholangiocyte organoids [12, 13, 46].

In light of all these advantages, it is important to point out that one of the hallmarks of cAMP is that it both activates and inhibits cell proliferation [109]. For hepatocytes, researchers have also demonstrated the dual effects of cAMP [110, 111], which stimulates hepatocyte proliferation in G0 or early G1 phase and specifically inhibits DNA synthesis in late GI phase [110]. Therefore, FSK may have a “two-sided” effect on hepatocyte proliferation and liver organoid formation, and further investigation is needed.

#### FGFs

FGFs, which were first identified by Armelin in pituitary extracts [112], belong to a large family of growth factors comprising 23 members [113]. In addition to mediating the well-known processes of angiogenesis, wound healing, and metabolic regulation via paracrine or endocrine signaling [114–116], FGFs play important roles in processes downstream of embryogenesis, such as somitogenesis [117] and organogenesis [118]. There are four FGF receptors: FGFR1, FGFR2, FGFR3, and FGFR4 [119]. Various evidence has proven that FGFR signaling is essential for hepatocyte proliferation, differentiation, and liver regeneration [120–122], which makes FGFs potentially useful for the establishment of liver models.

It has long been appreciated that FGF signaling is essential for liver specification, a previous study demonstrated that FGF10 accelerated liver regeneration after acute liver injury and promoted the expansion of various cells in the liver, including HPCs [123]. Therefore, it is not rare for scientists to use FGF10 in the culture of liver organoids. As an example, Huch added FGF10 to the EM used to establish liver progenitor culture when forming mouse liver organoids from Lgr5 + stem cells [41]. In addition, there were some reports of using FGF7 together with FGF10 in EM [45, 46, 93]. Notably, both of these two factors belong to the FGF7 subfamily and activate the same FGFR2b receptor, which confers similar physiological functions [113]. However, we also noticed that Seon Ju Mun and his team eliminated FGF10 from their hepatic medium because of its non-necessity in organoid expansion and high costs, but they affirmed the indispensable role of FGF10 in overcoming the differentiation barrier [15].

In addition to their pro-proliferative effect, FGFs also play a role in hepatic differentiation. FGF signaling was reported to be critical for liver development during embryogenesis and to regulate the morphogenetic growth of the hepatic endoderm [124]. A moderate level of FGF signaling can facilitate the differentiation of the ventral foregut endoderm to a liver fate [125]. FGF19 is commonly applied to induce liver organoid differentiation. A previous study chose human DM containing FGF19 to differentiate organoids into hepatocyte phenotypes, and both in vitro and in vivo analyses indicated the strong hepatocyte functions of the differentiated organoids [42]. In addition, another study switched to the final differentiation medium containing FGF19 in the last stage to derive a hepatic organoid containing functional liver parenchymal cell types, and immunofluorescence showed high expression levels of epithelial and hepatocyte markers [16].

Nevertheless, it was noted that the activating effect of FGFs on hepatocytes may also lead to hepatocellular carcinoma (HCC) development, and some FGFs and their receptors are involved in tumor development and progression [126]. For example, overexpression of FGF10 in mice was reported to induce multifocal pulmonary adenoma formation [127]. Another study showed that FGF19 induced hepatocellular carcinoma although it promoted hepatocyte proliferation in the early stage [128]. These results suggest that FGFs might play a role in cancer development, which may limit their clinical application. The adverse effects of FGFs on liver organoids have not been thoroughly investigated, and in terms of the current studies, the potential benefits far outweigh these risks.

#### TNF-α

Tumor necrosis factor (TNF), a major inflammatory cytokine, was initially identified for its capacity to induce rapid hemorrhagic tumor necrosis [129]. It is considered a crucial mediator of cytokine networks as well as a major regulator of the inflammatory process [130]. Many studies have reported the beneficial effects of TNFα on hepatocyte proliferation.

Interleukin (IL)-6 and TNF-α are both important signals in the regenerative response after partial hepatectomy [131]. Some researchers have reported that a lack of TNF-α contributes to a delay in liver regeneration [132]. As inflammatory cytokines secreted by Kupffer cells, TNF-α and IL-6 play an essential role in the priming phase by which hepatocytes re-enter the cell cycle [83, 131] to enhance cell proliferation. It was reported that they exert such effects by activating a range of transcription factors (NF-kB, JAK/STAT, AP-1, and YAP) [133, 134].

Based on these positive effects, the innovative use of inflammatory cytokines, represented by TNF-α, in the establishment of liver 3D organoid cultures in vitro was innovatively reported by Peng et al. [44]. In their experiment, TNF-α or IL-6 was added into the traditional organoid EM, and they found that TNF-α promoted the formation of hepatocyte colonies. Amplified organoids in vitro exhibited active liver function such as albumin secretion, CYP3A11 enzymatic activity, low-density lipoprotein (LDL) uptake, and glycogen storage. Furthermore, after gradually removing TNF-α, hepatocyte expansion decreased, lipids accumulated, and eventually, deterioration occurred [44]. Unfortunately, another inflammatory cytokine, IL-6, is not a good alternative to TNF-α. IL-6 has been reported to promote crypt organoid proliferation [135], and the reason why it does not work well enough for hepatocyte organoids needs to be further explored.

However, a different opinion about the application of TNF-α was reported in a more recent study from the Huch group. The TNF-α-free medium formulation was followed, and the authors concluded that the role of TNF-α could be replaced by FGF7, FGF10, and RSPO1-CM [46]. In addition, although the adverse effects of TNF-α on organoids have not yet been reported, there are some reasons for concern. (i) Direct hepatotoxicity: TNF is known to independently mediate murine hepatocyte apoptosis and subsequent liver failure [136, 137]. (ii) Carcinogenicity: TNF-α exerts proliferative effects via the upregulation of transcription factors in hepatocytes; these transcription factors include NF-kB, whose overexpression is linked to the development of hepatocellular carcinoma [138]. Other potential mechanisms underlying the cancer-promoting role of TNF have also been summarized before [139]. Together, this evidence shows the risks of applying TNF-α in organoid culture.

In conclusion, despite these reports of beneficial or detrimental effects of TNF-α, there is a lack of additional evidence on the use of TNF-α in hepatocyte organoid culture, and more attempts are needed.

### Differentiation medium

After cells form spheroids in EM, organoids can be passaged for long-term culture or, alternatively, further differentiated. Cholangiocyte organoids hardly express mature hepatocyte markers when formed in EM [41], and differentiation is necessary for them to acquire functional hepatocyte characteristics, thus enhancing the transplantation efficacy to support liver functions. As for hepatocyte organoids, DM is also defined because of the connection between transplantability and hepatocyte maturity [45]. It is worth mentioning that, in addition to the DM we will further introduce in this paper, cholangiocyte organoids have also been reported to form functional branching cholangiocyte organoids with tubular structures resembling the bile duct trees under particular culture conditions in vitro [140].

As shown in Table 3, the differentiation approaches reported in recent publications have varied, and we noticed that researchers often choose to remove or add some components in their own EM. This may explain certain differences in the use of gastrin I, TNF-α, EGF, CHIR99021 and some additives in DM between studies.

**Table 3:** Detailed components of different differentiation media

Origin	2013 Huch et al. [41]	2015 Huch et al. [42]	2016 Broutier et al. [12]		2018 Peng et al. [44]	2018 Hu et al. [45]	2020 Sorrentino et al. [13]		2020 Gómez-Mariano et al. [14]
	Mouse	Human	Mouse	Human	Mouse	Human	Mouse	Human	Human
Type	Cholangiocyte organoid	Cholangiocyte organoid	Cholangiocyte organoid	Cholangiocyte organoid	Primary hepatocyte organoids	Fetal hepatocyte organoids	Cholangiocyte organoid	Cholangiocyte organoid	Cholangiocyte organoid
Basal medium	AdDMEM/F12	AdDMEM/F12	AdDMEM/F12	AdDMEM/F12	William’s E medium	AdDMEM/F12	AdDMEM/F12	AdDMEM/F12	AdDMEM/F12
HEPES (mM)	/	/	10	10	/	√	/	/	10
GlutaMax	/	/	1%	1%	1%	√	/	/	1%
Antibiotics	/	/	Penicillin–Streptomycin: 1%	Penicillin–Streptomycin: 1%	Penicillin–Streptomycin: 1% Normocin: 0.2%	√	/	/	Penicillin–Streptomycin: 1%
Non-essential amino acids	Contained in AdDMEM/F12	Contained in AdDMEM/F12	Contained in AdDMEM/F12	Contained in AdDMEM/F12	1%	Contained in AdDMEM/F12	Contained in AdDMEM/F12	Contained in AdDMEM/F12	Contained in AdDMEM/F12
B-27	√	1%	2%	2%	2%	√	√	√	2%
N_2_	√	1%	/	1%	1%	/	√	√	1%
N-acetylcysteine (mM)	1.25 μM	/	1	1	1.25	1.25	1.25 μM	1.25 μM	1
Rspo1	/	/	/	/	/	RSPO1 CM^a^: 15%	/	/	/
Nicotinamide (mM)	/	/	/	/	10	10	/	/	/
Gastrin I (nM)	10	10	10	10	/	10	10	10	10
EGF (ng/ml)	50	50	50	50	25	50	50	50	50
HGF (ng/ml)	/	25	/	25	50	50	/	/	/
FGFs (ng/ml)	FGF10: 100	FGF19: 100	FGF10: 100	FGF19: 100	/	FGF10: 100 FGF7: 100	FGF10: 100	FGF19: 100	FGF10: 100
CHIR99021 (μM)	/	/	/	/	3	3	/	/	/
A83-01 (μM)	0.05	0.5	0.05	0.5	1	2	0.05	0.05	0.05
OSM (ng/ml)	/	/	/	/	/	10	/	/	/
TNFα (ng/ml)	/	/	/	/	100	/	/	/	/
Dexamethasone (μM)	30^b^	30	3^b^	3	3	1	3^b^	3^b^	/
DAPT (nM)	10	10 μm	10 μm	10 μm	/	/	10	10	10 μm
BMP7 (ng/ml)	/	25	/	25	/	/	/	25	/

^a^Conditioned medium (CM)

^b^Treatment during the last 3 days of differentiation

Beyond that, some of the constituents previously present in EM have changed. As a Wnt signaling agonist, the promoting effect of R-spondin 1 on hepatocyte proliferation is undisputed [141]. However, Laura Broutier and his team noted that the removal of R-spondin 1 induced organoids to differentiate toward a hepatocyte fate [12]. Among the reports included in our statistical analysis, the Hu group is the only one that did not explicitly mention the removal of R-spondin 1 from DM [45]. HGF is also a well-known mitogen that stimulates DNA synthesis in hepatocytes [80]. In some reports, pro-proliferative factors such as HGF and nicotinamide were added to establish liver progenitor cultures, but these factors were no longer included in the DM [12–14, 41, 42], probably because their pro-proliferative effects are no longer indispensable at this stage.

Differences also exist in some ingredients that are unique to the DM. Notch inhibitors (such as FGF19, dexamethasone, and DAPT), potent ductal-morphogenesis inducers [142], are commonly applied to induce the cells to acquire a hepatocyte phenotype [45]. Glucocorticoid (represented by dexamethasone) has long been used in the differentiation of liver cells [91]. Although it present in most organoid DM formulations, dexamethasone was added only during the last 3 days of differentiation in some studies [13, 41, 98], but was used throughout the process in other studies [42, 44, 45].

In addition, a detailed summary of the usage of DAPT, BMP-7 and oncostatin M (OSM**)** is provided in the following sections.

#### DAPT

(N-[N-(3,5-diflfluorophenylacetyl)-l-propanoyl]-s-phenylglycine butyl ester (denoted DAPT), a γ-secretase inhibitor, is known to inhibit all four receptors of the Notch pathway [143]. As an evolutionarily conserved mechanism, Notch signaling is a powerful regulator of cell fate. In addition to its apoptotic and proliferative effects, Notch signaling acts as a crucial factor in cellular differentiation [144, 145]. Since Notch activation suppresses cellular differentiation to the next state [146], Notch inhibitors such as DAPT, BMP, and dexamethasone are commonly used to induce the differentiation of multiple cell types [147–149].

To date, the mechanism of DAPT in liver development has not been fully elucidated. A previous report demonstrated that inhibition of the Notch pathway by DAPT promoted the differentiation of fetal liver stem/progenitor cells (FLSPCs). In this experiment, DAPT-induced FLSPCs showed similarities to mature hepatocytes in terms of cellular morphology, markers, and functions [92]. Interestingly, the hepatic differentiation-promoting effects of Notch inhibitors in turn suppressed differentiation toward the cholangiocyte fate, and differentiated cells exhibited upregulation of hepatic biomarkers with downregulation of bile duct markers [150]. This was also confirmed by a later study, data showed that inhibiting cholangiocyte differentiation indirectly promoted hepatocyte differentiation in the dominant state, and the detailed mechanism of Notch-mediated regulation probably depends on HNF-1β (the downstream factor of Notch) [151].

Thus, DAPT is now widely included in DM for liver organoids generated from primary tissue both from humans and mice [12, 13, 41, 42], as well as during the terminal stages of differentiation of PSC-derived organoids [16, 28]. In a study from the Huch group, as an example, the authors established a detailed protocol for the generation of self-renewing 3D organoids from adult liver cells and performed genetic manipulation experiments as well. In this article, 10 µM DAPT was added to the basal medium to obtain mouse and human liver DM [12]. In addition, the Yuan Guan group supplemented DM with pro-differentiation factors including DAPT during the final stage of organoid culture, and immunostaining revealed that the parenchymal organoids formed during this phase highly expressed ALB, CK8, and A1AT, indicating their differentiation into functional mature hepatic organoids [28].

To our knowledge, DAPT also has other functions, such as anti-inflammatory effects [152] and suppressing the deterioration of various tumor types, including liver cancer [153, 154]. Recent studies have not demonstrated its negative impact on liver organoids. Overall, DAPT is a promising ingredient in DM that promotes the differentiation of expanded organoids toward a hepatocytic fate rather than a bile duct fate by inhibiting the Notch signaling pathway. There are some exceptions to this observation, and we will later discuss the possible relationship between DAPT and another important pro-differentiation factor, the OSM.

#### BMP-7

Affiliated the TGF-β superfamily [155], BMPs have long been applied in bone formation and have shown a strong ability to enhance bone regeneration in the context of fracture nonunion, spine surgery, and Oral & Maxillofacial Surgery [156, 157]. In recent years, some members of the BMP family have been recognized as multifunctional cytokines that can mediate the growth and differentiation of many other cell types [158–160].

There are more than 20 members of BMP family [161], and evidence suggests that the liver is an important target for BMPs [124]. In addition to its proliferative effects on hepatocytes, BMP signaling is essential for liver specification [124]. Hikaru Sugimoto and coworkers administered rhBMP-7 to mice after partial hepatectomy and found it facilitated liver regeneration by enhancing hepatocyte proliferation [81]. In addition, BMPs have been widely used for hepatic generation from PSCs due to their hepatic specification effects on the ESC-derived DE [82, 162]. Initially, based on its hepatocyte proliferation-promoting effect, Huch et al. added BMP-7 into the EM for human liver organoids, and they found that it also promoted the expression of hepatocyte markers (ALB and CYP3A4) [42]. Since then, BMP-7 has become a commonly used additive for the differentiation of tissue-derived liver organoids (Table 3).

In the protocol presented by Broutier, BMP-7 was included in human liver DM, and 60% of human liver cells were differentiated into ALB- and HNF4α-positive cells after the differentiation phase (11–14 days), exhibiting binucleation (considered a sign of mature hepatocytes) [12]. However, we noticed that while DAPT and dexamethasone were present in both mouse and human DM, BMP-7 was only present in human liver DM. This may be explained by the fact that in contrast to mouse cells, human liver cells require TGF-β signaling to achieve long-term culture [42].

However, even though there is no longer any doubt about the regulatory activities of BMPs in the liver, the underlying profibrotic and tumorigenic properties of BMPs may need to be further investigated [163]. (i) While BMP-7 has long been known to have antifibrotic properties in renal and pulmonary tissues [164, 165], its role in liver fibrosis is still controversial. Some studies have pointed out that BMP-7 induces the proliferation of hepatic stellate cells (HSCs, the main ECM-producing cells involved in liver fibrogenesis, whose excessive accumulation can lead to liver fibrosis and cirrhosis) [166]. In addition, upregulation of BMP was observed in blood from patients with chronic liver diseases and human liver cirrhosis tissues [166]. (ii) The clinical relevance of BMPs to HCC deserves special attention [167, 168]. BMP-4 and BMP-9 were overexpressed in human HCC tissues and promoted HCC progression. In a recent study, imbalance of the TGF-β1/BMP-7 pathway was found to be associated with the aggressiveness of HCC and was linked to adverse clinical outcomes [169].

In summary, BMP-7 is an indispensable component of DM for human liver organoids. Although can have many adverse effects on tumorigenicity and profibrotic properties, the unfavorable role of BMP-7 in liver organoid culture still requires further investigation. Furthermore, the roles of other BMPs in tissue-derived liver organoids has not yet been investigated, but it is tempting to speculate that other BMP ligands may also have a promoting effect on liver organoid proliferation and differentiation.

#### OSM

For human-derived liver organoids, there is another DM additive: OSM. As a cytokine produced by monocytes and activated T-lymphocytes, OSM has been reported to be similar to members of the IL-6-type cytokine family in terms of both structure and function [170, 171]. However, in addition to its common functions in inflammation as the IL-6-type cytokine family, OSM also plays a role in promoting fetal liver development.

As early as 1999, Akihide Kamiya and his team found that OSM can induce the maturation of fetal hepatic cells in combination with glucocorticoids, which was proven by the expression of hepatic differentiation markers, glycogen accumulation, and a more mature morphology [172]. Subsequent studies further explored the underlying mechanism, demonstrating that OSM can be produced by hematopoietic cells in the mid-fetal liver and expand in a paracrine manner to induce the maturation of fetal hepatocytes in this stage. They also determined that OSM induces the expression of fetal hepatic differentiation markers via the STAT 3 pathway [84].

Based on these findings, a new differentiation medium for human fetal hepatocyte organoids containing OSM and dexamethasone was defined in an influential study [45]. In this study, fully differentiated liver organoids induced with OSM exhibited faster expansion and proliferation rates than undifferentiated cells when transplanted into the damaged mouse liver and subjected to long-term cultivation [45]. Notably, although OSM has been applied in only one particular report for the differentiation of fetal hepatocyte organoids thus far [45], it has been widely used in the final induction stage of PSC-derived liver organoids (as mentioned above, the principles and methods used in PSC-derived organoids at this stage of differentiation are similar to those used in tissue-derived organoids) [15, 17, 28].

However, although it was originally described as an anticancer agent, OSM was recently found to promote tumor progression in some cancers, such as HCC [173]. After treatment with OSM, the HepG2 cell line exhibited high GP73 expression (a biomarker of HCC), and the serum of patients with HCC and cirrhosis showed higher OMS levels than the control group, indicating a close relationship between OSM and liver diseases [173]. Additionally, the role of OSM in liver fibrosis is still under debate. On the one hand, the overexpression of OSM led to a rapid progression of liver fibrosis in mice [174], as well as increased collagen production in human hepatic stellate cells [175], with strong profibrotic effects. On the other hand, there is also evidence for a protective role of OSM in other fibrotic experimental models. For instance, in a rat model of dimethylnitrosamine (DMN)-induced liver fibrosis, OSM gene therapy alleviated liver damage by reducing hepatocyte apoptosis and fibrosis as well as promoting proliferation [176].

In addition, it is worth pointing out that OSM has not been used together with DAPT (Table 3), probably because of their similar effects on liver organoid differentiation. Although we failed to determine whether the two ingredients have conflicting mechanisms by which they promote liver organoid differentiation, some studies noted a positive feedback loop between the Notch pathway and STAT3. Suppressing Notch signaling with DAPT resulted in reduced *E. coli*-stimulated phosphorylation of STAT3 [177] (STAT3 is the core machinery by which OSM exerts its effects on hepatic differentiation). In addition, an activating effect of Notch on IL-6 has also been reported in breast cancer [178, 179]. This evidence suggests a possible mechanistic conflict between OSM and DAPT. The exception was that a previous study added OSM alone during first stage of differentiation while DAPT was accessed in the second stage for the cultivation of iPSC-derived hepatic organoids, and those two factors had synergistic facilitation effects, leading to more mature organoids [28]. Since direct evidence is still lacking, whether these factors can be used in combination awaits further investigation.

As discussed above, OSM promotes human liver organoid differentiation, but this has not been observed in mouse-derived liver organoid cultures. We subsequently noticed that some studies pointed out that the receptor systems for OSM in mice are different from those in humans [180, 181], which may explain its confined application only in human-derived liver organoids. In addition, OSM plays a major role in hepatic maturation during the middle and late fetal periods of liver development, but its expression starts to significantly decrease during the late fetal and neonatal stages [84]. This may explain why OSM is used only in the DM for primary fetal human hepatocyte organoids instead of adult hepatocytes.

## Future perspective and conclusion

Although much progress has been made in the field of organoid development, there are still some limitations that impede the extensive application of liver organoids (e.g., insufficient cell maturity, incomplete function, and restricted cell types) [182]. To address these problems, “multi-tissue organoids”, named in the consensus proposed by Ary Marsee et al. [4], have attracted much attention. This type of organoid system requires coordination of both parenchymal and supporting cells. The non-parenchymal cells in the liver (NPCs, including hepatic stellate cells, Kupffer cells, sinusoidal endothelial cells, etc.) have been proven to play a crucial role in liver tissue engineering [183], and a co-culture system of hepatocytes and NPCs in vitro was applied to modulate the phenotypic status of hepatocytes [184]. For instance, Rie Ouchi et al. induced a liver organoid model containing hepatic stellate cells and Kupffer cells via co-differentiation from human iPSCs [185]. Furthermore, endothelial cells were introduced into a 3D liver organoid culture system to address the fact that an excessive organoid size (larger than 3–4 mm) restricts the penetration of nutrients and oxygen into the center of the sphere [186], and the vascularized liver organoids showed improved cellular activity and liver function [187]. It was also reported that the incorporation of blood vessels and liver organoids may be indispensable for recreating the intricate microenvironment of complex liver diseases (such as primary liver cancer) [188] and simultaneously alleviating the cellular necrosis caused by ischemia in the center of the spheroid. However, protocols to support the self-renewal of multi-tissue organoids system are still lacking [4], and further work is necessary to determine suitable culture conditions for more widespread application. In addition, researchers are also exploring the culture patterns of “multi-organ organoids”, which is a highly intricate type of organoid induced from human PSCs and consist of several types of organs (i.e., hepato-biliary-pancreatic organoids) [11, 30].

In addition, different culture methods and media for organoids have been selected when considering the application scenario. (i) Disease models: In recent years, thanks to rapidly evolving technology, some groups have successfully constructed liver organoids that can reproduce some characteristics of steatosis or nonalcoholic fatty liver disease (NAFLD) from both tissue-resident cells and PSCs [93, 185, 189–191]. For example, Delilah Hendriks pointed out that medium components influenced the steatosis phenotype; RSPO1-conditioned medium, and B27 supplement were removed, and William’s Medium E +  +  + instead of AdvDMEM +  +  + was chosen as the basal medium [93]. (ii) Therapeutic transplantation: Following hepatocytes, hepatic progenitors, and NPCs, liver organoids became a reliable source for transplantation [192]. The main challenge for organoid-based transplantation therapy is determining how to transform research experiments into clinical applications. Since portability is usually thought to be connected to hepatocyte maturity [45], efforts have been made to promote the maturity of transplantable liver organoids, and the components of DM continue to evolve (Table 3). (iii) Drug screening: Liver organoids have shown both accuracy and high efficiency in drug validation and testing for benign and malignant liver diseases [193]. However, the influence of the culture medium composition should be considered cautiously when organoids are applied in the context of drug validation or toxicity assessment. For example, Rie Ouchi treated human liver organoids (HLOs) with FGF19 to verify the effects of FGF19 on Wolman disease, but it is well known that the FGF family (including FGF19) plays an important role in the culture of liver organoids, and its impact on the experimental results is largely unknown [185].

**Fig. 3:**
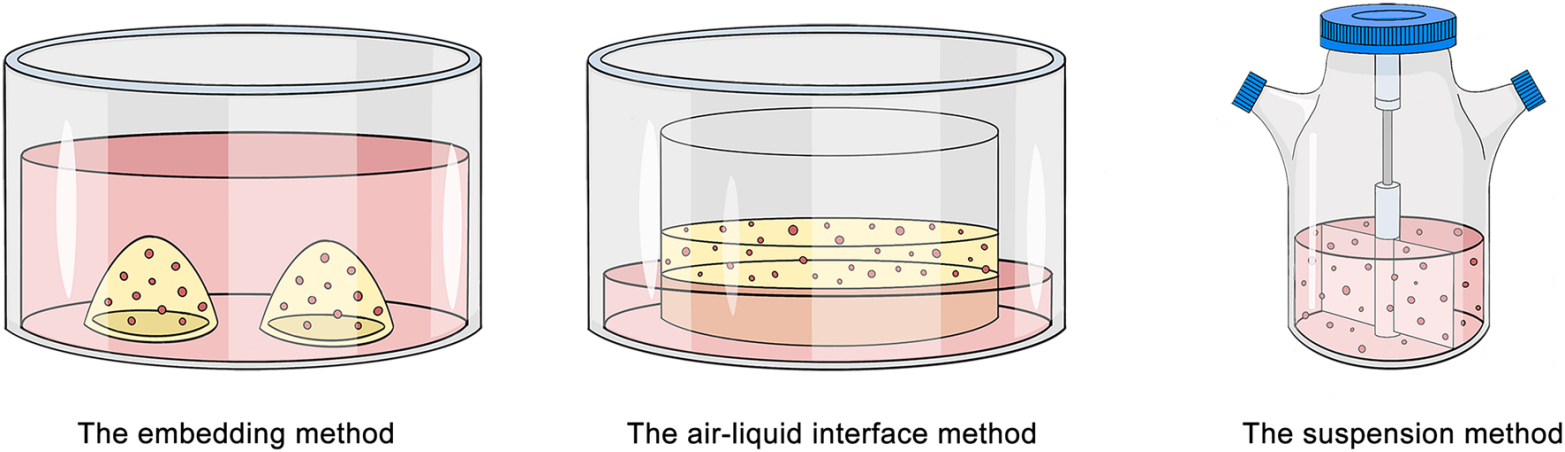
Three mainstream culture techniques for organoids

Some critical questions related to 3D culture techniques remain. The embedding method (which is performed as we have described above) is currently the most widely used liver organoid culture method [182], in this approach, solid ECM (such as Matrigel) promotes cell growth and the 3D characteristics of organoids. Of course, other strategies, such as the air–liquid interface (ALI) method and the suspension method, exist (Fig. 3). The ALI method was first applied in 3D culture by Calvin J. Kuo Lab, cells were grown on a thin microporous membrane, and the culture medium was only in contact with the basal side of the membrane [194]. This approach has become a strategy for the generation of kidney [195], brain [196], and gastrointestinal [197] organoids. Compared to submerged cell culture systems, the ALI system provides a higher oxygen concentration, and cells cultured in ALI exhibit enhanced cell–cell interactions and cell-stimulant interactions [198]. Therefore, ALI culture system comparatively enables accelerated organoid formation, as well as improved oxygenation of different types of organoids [199, 200]. Additionally, James T Neal and colleagues have demonstrated the unique role of the ALI method in the precise replication of complex original organ structures and immune micro-environment [201]. In their study, patient-derived tumor organoids preserved the intricate architectures of tumor parenchyma and stroma, including functional tumor-infiltrating lymphocytes. [201]. However, a disadvantage of ALI is its susceptibility to contamination by microbial or fibroblast cells [198]. The suspension method was mainly used in optic cup organoids and some brain organoids, which allows the cells to develop in a suspended and scaffold-free environment [202, 203]. The continuous agitation in this dynamic culture system contributed to better absorption of oxygen and nutrients compared with the two static culture methods mentioned above [204].

Worth mentioning that the clinical application of organoids for transplantation or high-throughput drug screening requires large-scale organoid production [205, 206]. A recent study described a spinner flask-based suspension method for the mass production of human adult stem cell-derived liver organoids, LGR5-positive liver stem cells were expanded with high efficiency and further differentiated into functional liver organoids [207]. Additionally, Takanori Takebe et al. proposed a unique method based on an omni-well-array culture platform for large-scale production of iPSC-derived liver buds [208]. However, studies have indicated that clinical improvement requires the transplantation of more than billions of cells [209], surpassing the capabilities of these current advancements. In addition, the inclusion of Matrigel in the medium of dynamic suspension culture remains a limitation for clinical applications. Therefore, replacing Matrigel in the spinner flasks with a suitable hydrogel is believed to be one of the future directions [207]. In conclusion, the rapid and large-scale culture of liver organoids holds great promise, but further extensive efforts will be needed in the future.

In addition, several factors, such as neurotransmitters, bile acids, insulin, and hedgehog, are already considered molecular signals during liver regeneration [83], but their effects in liver organoid culture have not been investigated. (i) Norepinephrine: In addition to being produced by cells of the sympathetic nervous system and the adrenal medulla, norepinephrine can also be produced by hepatic stellate cells [210]. This hormone excites the production of HGF and EGF and enhances their mitogenic effects [211, 212]. (ii) Serotonin: Serotonin can promote hepatocyte proliferation after partial hepatectomy through VEGF [213], which probably contributes to the ability of VEGF to increase HGF levels [214] and ultimately promotes hepatocyte proliferation indirectly. (iii) Bile acids and insulin: Positive impacts of bile acids [215] and insulin [216] on the ability to regulate hepatic metabolism and promote hepatocellular proliferation have been reported. These molecular signals, if proven to be able to facilitate organoid evolution, may lead to the development of novel medium components in the future. In addition, other inflammatory cytokines and other members of the FGF family or BMP family warrant further exploration.

Combining the above discussion with our own practical experience, we are inclined to believe that the following ingredients in EM are essential: R-spondin 1, nicotinamide, gastrin I, EGF, HGF, noggin (for cholangiocyte organoid), Wnt 3a (for cholangiocyte organoid), Y-27632, CHIR99021 (for hepatocyte organoid), and A83-01(for human origin), the absence of which may significantly influence the success rate of liver organoid formation. Otherwise, some of the ingredients, such as FGF7, TGFα, TNFα, and FSK, may be relatively non-essential. As for DM, the removal of R-spondin 1 and the addition of dexamethasone are quite necessary, while the removal of nicotinamide and HGF remained to be discussed, and the usage of FGFs, A83-01, OSM, DAPT, and BMP-7 can be flexible and adjustable. The precise effects of these controversial factors on liver organoids have not been thoroughly explored, and further control experiments are needed to enhance our understanding and optimize the utilization of these factors. Ideally, the removal of some non-essential factors may considerably reduce the cost of organoid culture. In addition, when determining the optimal liver organoid culture method, it is essential to consider the effectiveness, cost, and clinical safety collectively. Specifically, in the context of clinical transplantation practices, factors with potential tumorigenic or profibrogenic effects such as FGFs, TNF-α, BMP-7, OSM, and Matrigel should be excluded or substituted.

In conclusion, liver organoids are promising tools that play unprecedented roles in a wide range of biomedicine applications, and the culture strategy for liver organoids has been continually improved. However, more efforts are needed to establish a standard organoid culture system. Here, we summarize the liver organoid culture methods and different medium components of tissue-derived liver organoids, hoping to further promote the standardization and commercial application of liver organoids.

## Data Availability

All data used in this study are public.

## Abbreviations

ALI: Air–liquid interface
BMP: Bone morphogenetic protein
CM: Conditioned medium
cAMP: Cyclic adenosine monophosphate
DE: Definitive endoderm
DM: Differentiation medium
DMN: Dimethylnitrosamine
ESCs: Embryonic stem cells
EHS: Engelbreth-Holm-Swarm
EGF: Epidermal growth factor
ECM: Extracellular matrix
EM: Expansion medium
FLSPCs: Fetal liver stem/progenitor cells
FGFs: Fibroblast growth factors
FSK: Forskolin
FDA: Food and Drug Administration
HE: Hepatic endoderm
HPCs: Hepatic progenitor cells
HSCs: Hepatic stellate cells
HCC: Hepatocellular carcinoma
HGF: Hepatocyte growth factor
HLOs: Human liver organoids
IHs: Immature hepatocytes
iPSCs: Induced pluripotent stem cells
ICM: Inner cell mass
IL: Interleukin
LDL: Low-density lipoprotein
MHs: Mature hepatocytes
NAFLD: Nonalcoholic fatty liver disease
NPCs: Non-parenchymal cells
OSM: Oncostatin M
PSCs: Pluripotent stem cells
PFG: Posterior foregut
PEG: Polyethylene glycol
ProliHHs: Proliferating human hepatocytes
PGs: Prostaglandins
ROCK: Rho-associated kinase
TGF: Transforming growth factor
TNF: Tumor necrosis factor
TNFα: Tumor necrosis factor-α
2D: Two-dimensional
3D: Three-dimensional
